# Rapid Generation of Pulmonary Organoids from Induced Pluripotent Stem Cells by Co-Culturing Endodermal and Mesodermal Progenitors for Pulmonary Disease Modelling

**DOI:** 10.3390/biomedicines11051476

**Published:** 2023-05-18

**Authors:** Adam Mitchell, Chaowen Yu, Xiangjun Zhao, Laurence Pearmain, Rajesh Shah, Karen Piper Hanley, Timothy Felton, Tao Wang

**Affiliations:** 1 Division of Evolution, Infection and Genomics, School of Biological Sciences, Faculty of Biology, Medicine and Health, The University of Manchester, Manchester M13 9PL, UK; adammitchell1985@hotmail.co.uk (A.M.); chaowenyu@hospital.cqmu.edu.cn (C.Y.); xiangjun.zhao@postgrad.manchester.ac.uk (X.Z.); 2 Children’s Hospital of Chongqing Medical University, Chongqing 400014, China; 3 Division of Diabetes, Endocrinology & Gastroenterology, Wellcome Trust Centre for Cell-Matrix Research, School of Medical Sciences, Faculty of Biology, Medicine and Health, The University of Manchester, Manchester M13 9PL, UK; laurence.pearmain@manchester.ac.uk (L.P.); karen.piperhanley@manchester.ac.uk (K.P.H.); 4 Manchester University Hospital NHS Foundation Trust, Wythenshawe Hospital, Southmoor Road, Manchester M23 9LT, UK; rajesh.shah@mft.nhs.uk; 5 Division of Infection, Immunity and Respiratory Medicine, The Lydia Becker Institute of Immunology and Inflammation, Faculty of Biology, Medicine and Health, The University of Manchester, Manchester M13 9PL, UK

**Keywords:** pulmonary organoids, induced pluripotent stem cells (iPSCs), anterior foregut endoderm, mesoderm, alveoli epithelial cells, SARS-CoV-2, iPSC disease modelling

## Abstract

Differentiation of induced pluripotent stem cells to a range of target cell types is ubiquitous in monolayer culture. To further improve the phenotype of the cells produced, 3D organoid culture is becoming increasingly prevalent. Mature organoids typically require the involvement of cells from multiple germ layers. The aim of this study was to produce pulmonary organoids from defined endodermal and mesodermal progenitors. Endodermal and mesodermal progenitors were differentiated from iPSCs and then combined in 3D Matrigel hydrogels and differentiated for a further 14 days to produce pulmonary organoids. The organoids expressed a range of pulmonary cell markers such as SPA, SPB, SPC, AQP5 and T1α. Furthermore, the organoids expressed ACE2 capable of binding SARS-CoV-2 spike proteins, demonstrating the physiological relevance of the organoids produced. This study presented a rapid production of pulmonary organoids using a multi-germ-layer approach that could be used for studying respiratory-related human conditions.

## 1. Introduction

Induced pluripotent stem cells (iPSCs) are characterised by their capacity for indefinite self-renewal and their ability to differentiate into any of the mature cell types found in the adult human body. This includes differentiation to a pulmonary epithelial lineage such as alveolar cells [1]. Such differentiation schemes often mirror the embryogenesis of the cell or tissue type in question. In the case of alveolar cell development, this involves differentiation through definitive endoderm, anterior foregut endoderm (AFE), pulmonary progenitors and finally alveolar cells [2,3,4]. Often, such differentiation schema were developed in simple 2D culture systems with growth factors and small molecules used to stimulate or inhibit specific cell signalling pathways. However, there is increasing appreciation that stem cell fate can be determined by a myriad of other parameters including the cellular microenvironment, extracellular matrix, 3D spatial arrangement, mechanical cues including fluid flow, and the existence of multiple cell types [5]. This has led to the development of increasingly complex stem cell culture systems such as organoids or tissue matrix recellularisation [6,7].

In perhaps the first report on generating pulmonary organoids from iPSCs, Gotoh et al. demonstrated the production of pulmonary organoids using NKX2.1^+^ (a marker of early pulmonary lineage commitment) ‘ventralised’ AFE cells cultured within Matrigel also containing human foetal lung fibroblasts [8]. The approach to 3D culture was based on previous studies using primary pulmonary cells to generate organoids in vitro [9] but the study resulted in two important findings: the need for a pure endodermal population to generate lung epithelial cells and the requirement for a mesodermal population of cells to support that differentiation. In a series of papers, Dye et al. created ‘patterned lung organoids’ using anterior foregut endoderm spheroids [10,11]. They noted that whilst their AFE spheroids were 85–95% pure, a population of mesodermal cells persisted within and contributed to differentiation. Based on this, the authors were able to refine the growth factors necessary to generate mature pulmonary organoids. A more recent study also demonstrated that mesenchymal stem cells (MSC), when included in lung progenitor 3D cultures, improved alveolar differentiation of iPSC-derived cells, and MSC-conditioned medium alone was sufficient to promote alveolar organoid formation [12]. Taken together, these studies underline the importance of multi-germ-layer involvement in organoid development; however, the mesodermal component was poorly defined and protocols used for making alveolar organoids from iPSCs were lengthy.

In the initial stages of pulmonary embryogenesis, primitive lung buds form from a cluster of NKX2.1^+^ cells located in the anterior foregut of the epithelial tube that ultimately gives rise to the trachea and oesophagus [2]. Surrounding these cells is a layer of mesoderm that expresses a range of growth factors, notably BMP4, FGF10 and WNT2, the absence of which inhibits lung bud maturation [13,14,15]. Consequently, many such growth factors can be found in culture medium designed to induce the differentiation of AFE to pulmonary cells. It has previously been shown that the transcription factor GATA4, localised to the developing pulmonary mesenchyme at the epithelial buds, is necessary for normal pulmonary development [16]. Furthermore, GATA4 could enhance the expression of FGF10 [17]. These findings suggest that an iPSC-derived GATA4^+^ mesoderm could be an appropriate cell population to support the differentiation of AFE to a pulmonary fate in vitro. Mesodermal cells are a common intermediary in the production of cardiomyocytes; indeed, cardiac mesoderm interacts with the pulmonary endoderm in utero. In an effort to increase the efficiency of iPSC differentiation to cardiomyocytes, Gong et al. refined the common BMP4-mediated differentiation of iPSCs to mesoderm to include the γ-secretase inhibitor DAPT [18]. Amongst their findings was that DAPT significantly increased the expression of mesodermal markers, including GATA4, after 6 days of differentiation. This timing is well aligned with the production of FOXA2^+^ AFE cells in vitro.

Based on our previous studies involving the differentiation of iPSCs to alveolar cells, and adapting the work of Gong et al., we sought to generate organoids via the co-culture of FOXA2^+^ iPSC-derived AFE and GATA4^+^ mesoderm encapsulated within Matrigel hydrogels. IPSC-derived organoid culture systems have become increasingly popular not just as means to study organogenesis but also to study and model disease and aid drug discovery. For example, Chen et al. introduced *HPS1* mutations in iPSCs that were used to generate pulmonary organoids to recapitulate pulmonary fibrotic disease [19]; and Suezawa et al. created a model of pulmonary fibrosis using human PSC-derived alveolar organoids and identified inhibition of ALK5 or blocking integrin αVβ6 as potential therapeutic options [20]. Infectious respiratory diseases, such as the recent COVID-19 pandemic caused by the SARS-CoV-2 infection, are highly prevalent and can lead to extensive damage to the pulmonary epithelium. Several excellent studies have been published since the outbreak of the COVID-19 and some have identified candidate COVID-19 therapeutics [21,22,23,24]. The capacity to rapidly produce pulmonary organoids could be useful in understanding the pathogenicity of other respiratory viruses in the future. As such, we produced a method of generating pulmonary organoids by the co-culture of defined endodermal and mesodermal progenitors, and also sought to demonstrate that the organoids were capable of interacting with SARS-CoV-2 spike proteins to highlight their physiological utility.

## 2. Materials and Methods

Detailed materials and mathods are provided in Appendix A.

### 2.1. Cell Culture and Differentiation

Fully characterised iPSC lines (SERU7, SOJD3 and EIPL1) from the HipSci stem cell bank, Public Health England via ECACC, were maintained in Essential 8 Medium on Matrigel-coated plates. For AFE differentiation, iPSCs were dissociated with StemPro Accutase and then plated on Matrigel-coated plates at a density of 30,000 cells/cm^2^ in Essential 8 with 10 µM Y27632. The following day, the medium was changed to DMEM containing Glutamax, non-essential amino acids, human serum replacement 3, 5 µM CHIR99021 and 100 ng/mL Activin A, and this was termed day 0. On days 1–2, cells were cultured in the same medium minus CHIR99021. On day 3, cells were cultured in the same base medium with the addition of 1 µM Dorsomorphin and 2 µM SB431542. Finally, on day 4, cells were cultured in the same base medium with the addition of endo-IWR1 (200 nM) and SB431542 (2 µM). On day 5, AFE cells were characterised for phenotypic markers or used for pulmonary organoid construction. For mesoderm differentiation, iPSCs were plated on Matrigel-coated plates at a density of 60,000 cells/cm^2^ in Essential 8 with 10 µM Y27632. The following day, the medium was changed to DMEM containing Glutamax, non-essential amino acids, human serum replacement 3 and BMP4 (0.5 ng/mL), and this was termed day 0. On days 1–2, cells were cultured in the same base medium with the addition of Activin A (3 ng/mL), BMP4 (10 ng/mL) and FGF2 (5 ng/mL). Finally, on days 3–4, cells were cultured in the same medium but with the addition of 2 µM DAPT. On day 5, mesoderm cells were characterised for phenotypic markers.

For pulmonary organoid construction, 12 mm cell culture inserts were first coated with a 60 µL Matrigel. AFE and mesoderm cells were dissociated in TrypLE, and a cell suspension containing 500,000 cells each of AFE and mesoderm per organoid was mixed with an equal volume of Matrigel and 200 µL pipetted on to the first layer of Matrigel and set for 2 h at 37 °C, followed by adding a final 60 µL Matrigel and set for 2 h at 37 °C. Finally, DMEM containing Glutamax, non-essential amino acids, human serum replacement 3, BMP4 (10 ng/mL), FGF2 (10 ng/mL), FGF7 (10 ng/mL) and FGF10 (10 ng/mL) was added to the cell culture wells and inserts and incubated at 37 °C. All work was performed with cooled liquids and tips to prevent premature Matrigel polymerisation. Organoids were maintained in culture for 2–4 weeks and the medium changed every 2–3 days.

### 2.2. qRT-PCR

Total RNA from cells was extracted with a QIAGEN RNeasy Mini Kit and cDNA was synthesised using an iScript cDNA Synthesis Kit. Five ng cDNA per sample was used for standard qPCR with SYBR Green chemistry and pre-designed primers (Table A1). The expression was normalised to *GAPDH* or *B2M* and statistical significance determined by Student’s *t*-test.

### 2.3. RNA Sequencing

RNA sequencing (RNA-seq) was performed using total RNA derived from pulmonary organoids on an Illumina HiSeq 4000 sequencer. Detailed methods and data analysis are described in Appendix A.

### 2.4. Flow Cytometry

Cells were dissociated with Accutase, fixed in 4% PFA, and incubated with antibodies (Table A1) for cell surface proteins. Samples were then permeablised with 0.1% Triton X-100 followed by incubating with antibodies (Table A1) for intracellular proteins. After filtering through a 40 µm cell strainer, flow cytometry was performed on an LSR II Flow Cytometer. Data was analysed with FACSDiva software v8.0. (BD Biosciences, Wokingham, UK).

### 2.5. Confocal Imaging and Histology

Pulmonary organoids were harvested for immunofluorescent staining and confocal imaging 9 days after the mixing of AFE and mesoderm. Sample preparation was performed as described by Dekkers et al. [25]. After immunolabeling, the organoids were imaged on a Leica SP8 confocal microscope with 20× water immersion objective for multiphoton imaging. Details for the acquisition mode settings are listed in Appendix A. Human lung sections were obtained following informed consent and ethical approval (National Research Ethics Service, REC 20/NW/0302). Tissues were deparaffinised and rehydrated with xylene and ethanol followed by antigen retrieval and sequential incubation with the primary and secondary antibodies as listed in Table A1 in Appendix A. After dehydration with ethanol gradient, the samples were mounted with mounting media and imaged on an Olympus IX83 inverted microscope.

## 3. Results

### 3.1. Differentiation of iPSCs to Endodermal and Mesodermal Progenitors

**Figure 1:**
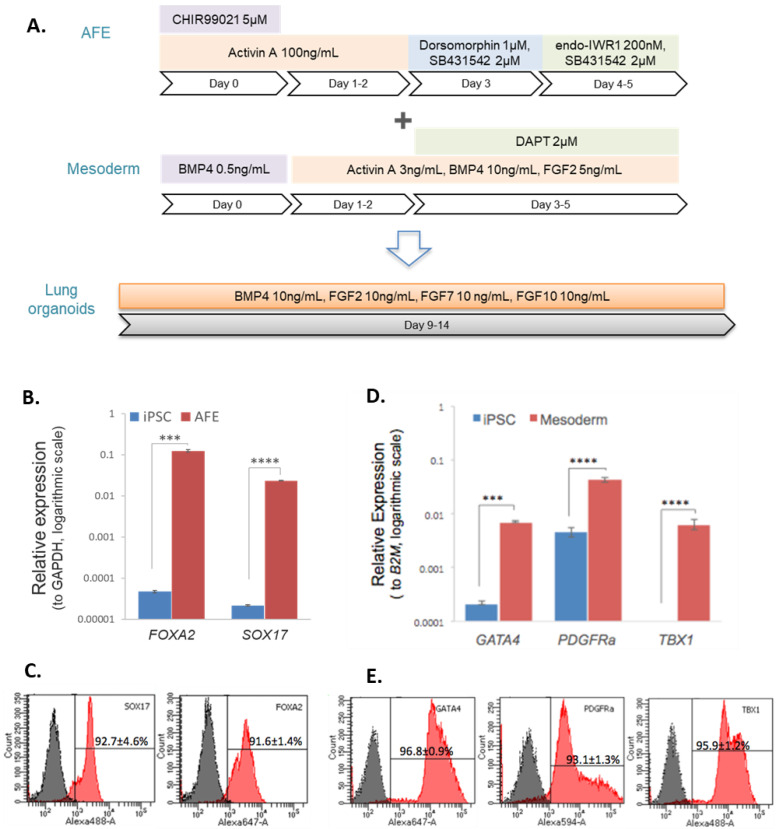
Characterisation of endodermal and mesodermal progenitors prior to co-culture. (**A**) Diagram illustrating the overall lung organoid differentiation process. (**B**,**D**) Gene expression analysis of iPSCs differentiated to AFE (**B**) and mesoderm (**D**) prior to co-culture. *n* = 3, data displayed as mean ± SEM. Statistical significance determined by unpaired *t*-test, *** *p* < 0.001, **** *p* < 0.0001. (**C**,**E**) Flow cytometry analysis of iPSCs differentiated to AFE (**C**) and mesoderm (**E**) prior to co-culture. Data displayed as representative histograms with the mean ± SEM, *n* = 3.

Prior to 3D pulmonary organoid construction, iPSCs were first differentiated to AFE and mesoderm (Figure 1A). After 5 days of endodermal differentiation, the expression of the endodermal markers *FOXA2* and *SOX17* was significantly increased in AFE cells compared with the undifferentiated iPSCs, *p* < 0.001 and *p* < 0.0001, respectively (Figure 1B). Across three different iPSC lines used in the study, the mean number of *FOXA2***^+^** and *SOX17*^+^ cells following differentiation was 92% and 93%, respectively (Figure 1C). After 5 days of mesodermal differentiation, the expression of the mesodermal markers *GATA4* and *PDGFRa* was significantly increased in the mesoderm cells compared with the undifferentiated iPSCs, *p* < 0.001 and *p* < 0.0001, respectively (Figure 1D). The expression of *TBX1* was detected in mesodermal cells whilst being almost absent in iPSCs. Flow cytometry analysis showed the mean numbers of *GATA4*^+^, *PDGFRa*^+^ and *TBX1*^+^ cells differentiated from the three different iPSC lines to be 97%, 93% and 96%, respectively (Figure 1E).

### 3.2. Spontaneous Pulmonary Organoid Formation by Co-Culture of Endodermal and Mesodermal Progenitors

**Figure 2:**
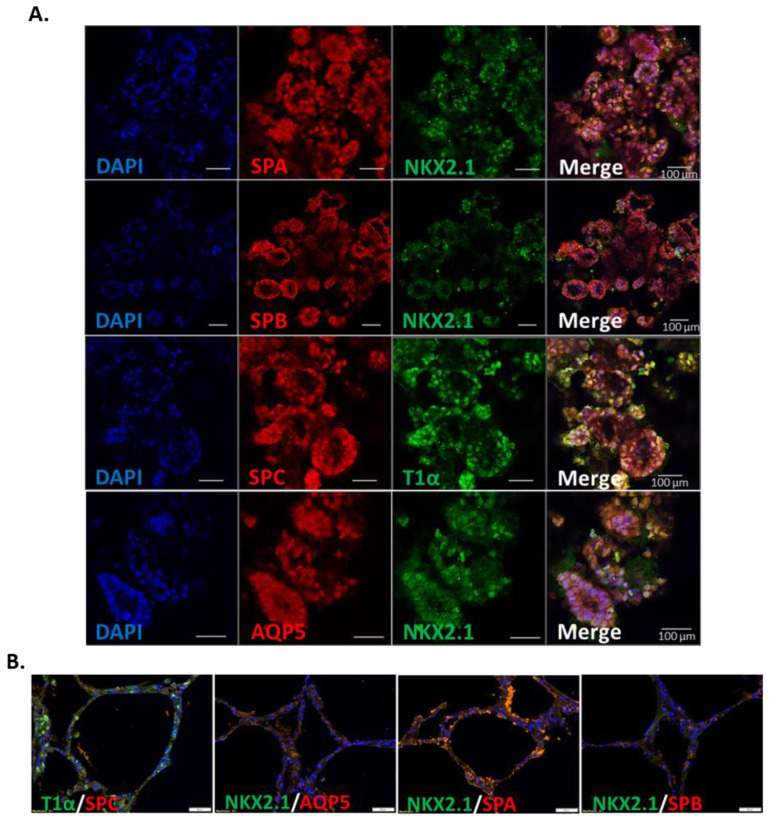
Immunofluorescent staining of alveolar cell markers in iPSC-derived pulmonary organoids. After the co-culture of AFE with mesoderm progenitors for 9 days, the organoids were stained for alveolar epithelial type I and type II markers including AQP5, T1α, SPA, SPB, SPC and NKX2.1. (**A**) Representative confocal images of organoids derived from 3 different iPSC lines. Scale bars 100 µm. (**B**) Staining of the same markers in normal human lung sections. Scale bars 50 µm.

**Figure A1:**
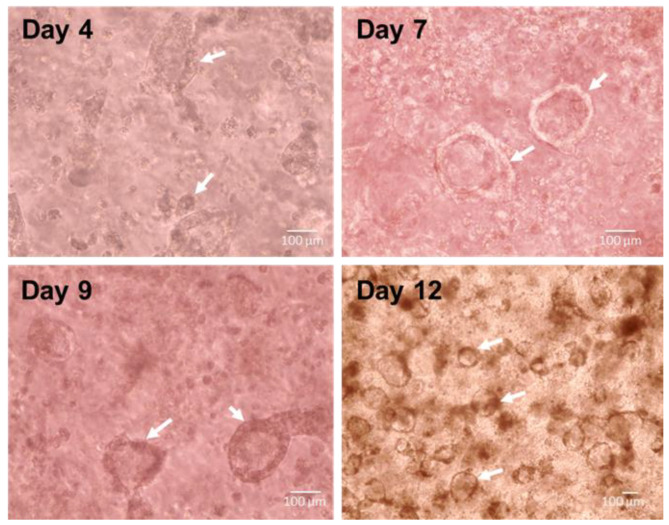
Images of pulmonary organoids during the course of differentiation. Pulmonary organoids were imaged under light microscope on day 4–12 days after co-culture of AFE and mesoderm progenitors. White arrows indicate organoids. Scale bars 100 µm.

**Figure A2:**
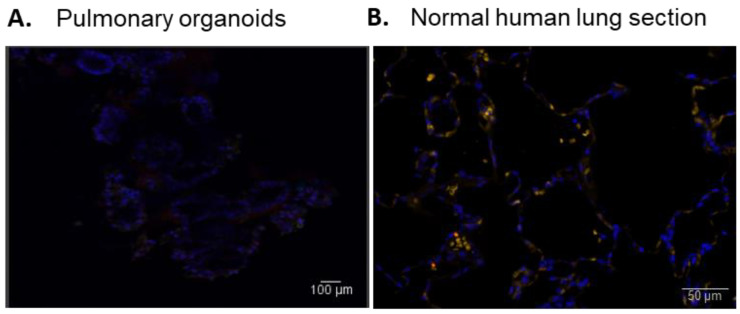
Negative control for immunofluorescent staining of alveolar cell markers in iPSC-derived pulmonary organoids. (**A**) After 14 days of co-culture of AFE and mesoderm progenitors, the organoids were stained with the secondary antibody only. Figure showed representative confocal images of organoids derived from 3 different iPSC lines and stained by AlexaFlour 594. Scale bars 100 µm. (**B**) The normal human lung section stained with secondary antibody AlexaFlour 594 only. Scale bars 50 µm.

AFE and mesoderm progenitors were co-cultured in a 1:1 ratio for 9–14 days in Matrigel to induce pulmonary organoid formation. During the process of differentiation, spherical pulmonary organoids were gradually formed which had a hollow internal lumen (Figure A1). AFE progenitors without co-culture with mesoderm cells could not form organoids. Immunofluorescent confocal microscopy revealed the existence of type II alveolar cell markers including surfactant protein A (SPA), surfactant protein B (SPB) and surfactant protein C (SPC) in the pulmonary organoids (Figure 2A), which were also shown in normal human lung sections (Figure 2B). Similarly, markers for the type I alveolar cells, including aquaporin 5 (AQP5) and podoplanin/T1α, were also confirmed in the pulmonary organoids and in the normal human lung sections (Figure 2A,B). The expression of NKX2.1 was weak in both the pulmonary organoids and normal human lung, suggesting a relative maturation of the organoids. Controls with secondary antibody staining were negative (Figure A2).

### 3.3. IPSC-Derived Pulmonary Organoids Express Lung-Specific Marker Genes

**Figure 3:**
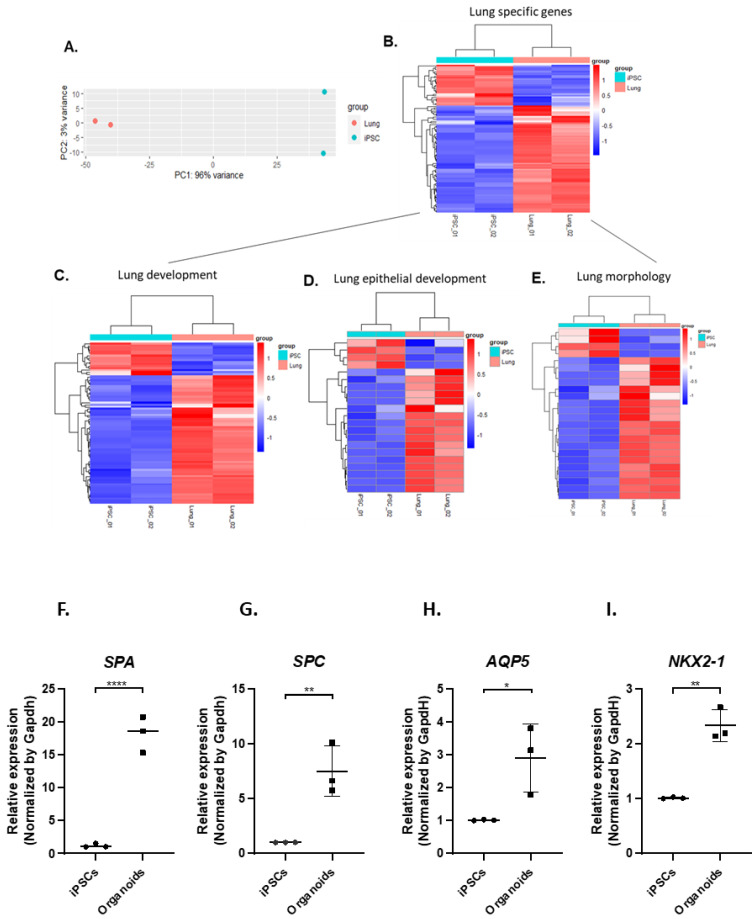
Gene expression profiling of iPSC-derived pulmonary organoids. Fourteen days into the pulmonary organoid generation following co-culturing of AFE and mesoderm progenitors, total RNA from the organoids and the undifferentiated iPSCs was analysed by RNAseq (**A**–**E**) and candidate genes were confirmed by RT-qPCR (**F**–**I**). (**A**) PCA plot comparing RNAseq data between organoids differentiated from 2 independent iPSC lines and the undifferentiated iPSCs. (**B**–**E**) Heatmaps showing differentially expressed genes relating to the lung development. (**F**–**I)** RT-qPCR for expression of alveolar epithelial type I and type II marker genes *SPA*, *SPC*, *AQP5* and *NKX2.1* from samples of 3 independent differentiations of 2 iPSC lines. Data are presented as mean ± SE, *n* = 3. Statistical significance was determined by an unpaired *t*-test, * *p* < 0.05, ** *p* < 0.01, **** *p* < 0.0001.

RNA sequencing (RNAseq) was then conducted on the pulmonary organoids differentiated from two different iPSC lines on day 14 after co-culture of AFE and mesoderm progenitors. Principle-component analysis (PCA) of the RNAseq data sets showed that the pulmonary organoids were clearly distinguished from the undifferentiated iPSCs (Figure 3A). A heatmap generated by DESeq analysis (Figure 3B) shows that a majority of the lung-specific genes were enriched in the pulmonary organoids compared to the undifferentiated iPSCs. Among the lung-specific genes, subsets of genes on the Gene Ontology (GO) terms of lung development, lung epithelial development and lung morphology were mostly upregulated (Figure 3C–E, Appendix C). The expression of key marker genes for type 1 (*AQP5*) and type II (*SPA*, *SPC*) alveolar cells, as well as lung-specific genes in general (*NKX2*-1), were validated by RT-qPCR (Figure 3F–I).

### 3.4. Pulmonary Organoids Express ACE2 Capable of Binding SARS-CoV-2 Spike Protein

**Figure 4:**
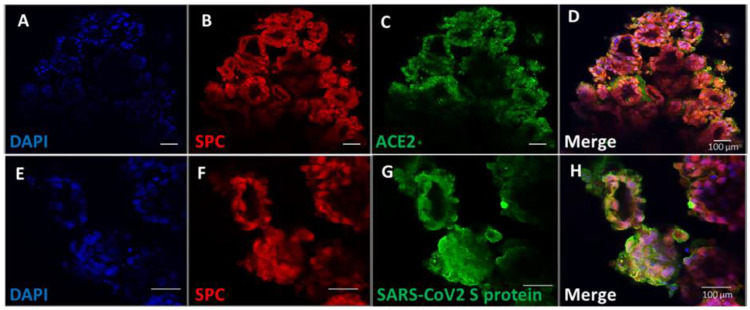
IPSC-derived pulmonary organoids express ACE2 capable of binding SARS-CoV-2 spike protein. Experiments were carried out on day 14 for pulmonary organoids post co-culture of AFE and mesoderm progenitors. (**A**–**D**) Organoids were immunostained for ACE2, as well as the alveolar epithelial marker SPC. (**E**–**H**) Organoids were incubated with recombinant SARS-CoV-2 spike protein followed by immunofluorescent staining using antibodies specific to the SARS-CoV-2 spike protein, as well as to the alveolar epithelial marker SPC. Figures are representative images of organoids derived from 3 different iPSC lines. Scale bars 100 µm.

To demonstrate the physiological relevance of the iPSC pulmonary organoids, their interaction with SARS-CoV-2 was investigated. It is known that the spike protein of SARS-CoV-2 binds the host angiotensin-converting enzyme 2 (ACE2) for viral entry into the host cells [26]. We demonstrated that ACE2 proteins were present in SPC-positive cells in the pulmonary organoids (Figure 4A–D). To determine the virus binding capability, recombinant SARS-CoV-2 spike proteins were incubated with the organoids and visualised by immunofluorescent microscopy. Results showed that there was clear detection of membrane-bound SARS-CoV-2 spike proteins on alveolar cells of the organoids (Figure 4G), and the staining was also co-localised to cells expressing SPC (Figure 4E–H).

## 4. Discussion

The aim of this study was to demonstrate a rapid production of pulmonary organoids via the co-culture of iPSC-derived endodermal and mesodermal progenitors, and to demonstrate that the organoids were capable of interacting with the respiratory virus SARS-CoV-2. Using a well-established approach for encapsulating cells in 3D hydrogels based on Matrigel, it was shown that pulmonary organoids spontaneously formed within 14 days of co-culturing endodermal and mesodermal progenitors. The resulting organoids expressed a range of proteins associated with type I and II alveolar cells. The pulmonary organoids also expressed ACE2 that was capable of binding the SARS-CoV-2 spike protein.

Previous studies demonstrated a clear need for a mesodermal component in establishing iPSC-derived pulmonary organoids [11,19], which is unsurprising given the role of mesoderm in driving pulmonary organogenesis. However, these studies utilised a mesoderm that was an unintentional by-product of endodermal differentiation. Based on the phenotype of pulmonary mesoderm in utero, we produced a GATA4^+^ mesoderm to support the differentiation of endodermal progenitors in vitro. Most of the current protocols in the literature use defined factors including CHIR and BMP4 to provide mesodermal cues for lung organoid differentiation [27,28]. Albeit successful, a few defined factors are unlikely to recapitulate the entire role that mesoderm plays during lung organogenesis. We have used a highly controlled approach to co-culture AFE and mesoderm progenitors. We found that the mesoderm progenitors are absolutely necessary for pulmonary organoid generation. AFE progenitors alone were not able to form organoids. Although we introduced an additional step for pulmonary organoid generation in the initial AFE and mesoderm differentiation, the protocols produce AFE and mesoderm progenitors in 5–6 days, which can be co-cultured directly for pulmonary organoid construction on Matrigel, without the need for selection or purification of the progenitors. The organoids started to form in 4 days after co-culture and robustly express lung-specific markers as early as day 9. Thus, the total protocol takes less than 20 days to produce lung organoids. This is much quicker than the existing published methods, which usually take 30–60 days [23,28], and is similar to the protocol used by Chen et al., which took 25 days [27].

**Figure A3:**
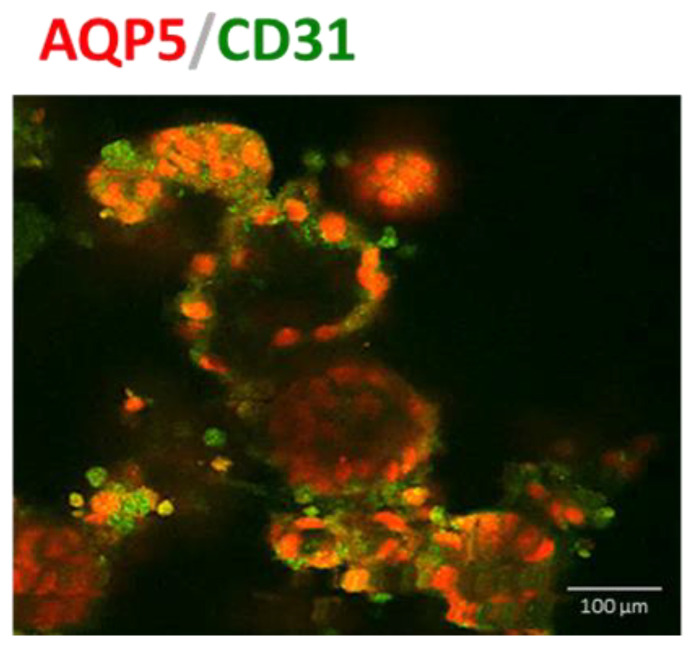
Immunofluorescent staining of endothelial marker CD31 in iPSC-derived pulmonary organoids. After 14 days of co-culture of AFE and mesoderm progenitors, the organoids were stained with endothelial cell marker CD31 (green) and co-stained with alveolar type I cell marker aquaporin 5 (AQP5, red). Scale bars 100 µm.

The mesoderm population is also desirable for the future production of more complicated lung organoids. By adding minimum further factors such as VEGF, this protocol has the potential to generate vascularised lung organoids. The vascular components would derive from the mesodermal population, as the growth factors employed in the protocol are very similar to the endothelial cell differentiation from iPSCs in our previous work [29]. In fact, we have already observed some expression of endothelial marker CD31 in the organoids surrounding the AQP5^+^ alveolar cells (Figure A3), suggesting the possible emergence of an endothelial population in the matrix of the organoids. Further work is required to understand the fate and contribution of the mesodermal progenitors to the organoid formation, and to optimise a protocol for the vascularisation of the lung organoids. Such an effort would potentially open the prospect for future studies of lung barrier function and modelling diseases under a more physiological context.

The expression of ACE2 receptor and subsequent interaction with the SARS-CoV-2 spike protein suggest that pulmonary organoids could in the future be used to study the interaction of respiratory pathogens with the pulmonary epithelium in vitro. A recent study by Porotto et al. demonstrated the infection of pulmonary organoids with parainfluenza virus localised to alveolar epithelial cells as would occur in vivo [30]. A more recent publication by Han et al. [21] attempted drug screening using pulmonary organoids to identify blockers of SARS-CoV-2 infection for the treatment of COVID-19. However, severe respiratory viral infections often cause extensive damage mediated by inflammatory cascades involving pro-inflammatory cytokines and alveolar macrophages and neutrophils. In order for pulmonary organoids to fully recapitulate the pathogenesis of respiratory viral infection, future work could incorporate iPSC-derived alveolar macrophages [31] and neutrophils [32], although considerable work is needed to understand the timing of introducing immune cells and how to maintain their phenotype over prolonged culture.

In summary, we have developed a multi-germ-layer approach for the rapid production of pulmonary organoids that has exhibited the capacity for infection by SARS-CoV-2, and with further development it may be useful in understanding the pathogenesis of future novel respiratory viruses and in pulmonary disease modelling.

### Limitations of the Study

One limitation of this study is that the organoids we differentiated were an alveolar model rather than an airway model. Although the alveolar-focused organoids derived from iPSCs have good values for future studies of a range of human conditions, it is worthwhile to further develop the protocol to build a full lung model. Additionally, we have aimed to develop a rapid protocol to produce the organoids, but we did not culture the organoids longer beyond the time point when specific alveolar cell markers were detected. Depending on the desired applications, a prolonged culture may benefit further maturation of the organoids.

## Data Availability

Data are all provided in this paper.

## Appendix A. Supplemental Information—Detailed Experimental Procedures

### Appendix A.1. Cell Culture and Differentiation

IPSC lines SERU7, SOJD3 and EIPL1 were supplied by HipSci stem cell bank, Public Health England, via ECACC. IPSC lines WT3 and WT4 were reprogrammed from adult human dermal fibroblasts, as reported in our previous publication [28]. IPSCs were maintained, feeder free, in Essential 8 Medium (Thermo Fisher Scientific, Loughborough, UK) and passaged at a 1:6 ratio, on Matrigel (Corning, Deeside, UK)-coated plates, every 3–4 days. For anterior foregut endoderm (AFE) differentiation, iPSCs were dissociated with StemPro Accutase (Thermo Fisher Scientific, Loughborough, UK) for 5 min, at 37 °C, then plated on Matrigel-coated plates at a density of 30,000 cells/cm^2^ in Essential 8 Medium with 10 µM Y27632 (Tocris, Bristol, UK). The following day, the medium was changed to Dulbecco′s Modified Eagle Medium (DMEM) containing Glutamax (Thermo Fisher Scientific, Loughborough, UK), non-essential amino acids (Sigma Aldrich, Gillingham, UK), human serum replacement 3 (Sigma Aldrich, Gillingham, UK), 5 µM CHIR99021 (Tocris, Bristol, UK) and 100 ng/mL Activin A (Peprotech, London, UK), and this was termed day 0. On days 1–2, cells were cultured in the same medium minus CHIR99021. On day 3, cells were cultured in the same base medium with the addition of Dorsomorphin (1 µM, Tocris, Bristol, UK) and SB431542 (2 µM, Tocris, Bristol, UK). Finally, on day 4, cells were cultured in the same base medium with the addition of endo-IWR1 (200 nM, Tocris, Bristol, UK) and SB431542 (2 µM). On day 5, anterior foregut endoderm cells were characterised for phenotypic markers or used for pulmonary organoid construction. For mesoderm differentiation, iPSCs were dissociated with StemPro Accutase for 5 min, at 37 °C, then plated on Matrigel-coated plates at a density of 60,000 cells/cm^2^ in Essential 8 Medium with 10 µM Y27632. The following day, the medium was changed to DMEM containing Glutamax, non-essential amino acids, human serum replacement 3 and BMP4 (0.5 ng/mL, Peprotech, London, UK), and this was termed day 0. On days 1–2, cells were cultured in the same base medium with the addition of Activin A (3 ng/mL), BMP4 (10 ng/mL) and FGF2 (5 ng/mL). Finally, on day 3, cells were cultured in the same medium but with the addition on DAPT 2 µM (Tocris, Bristol, UK). On day 5, mesoderm cells were characterised for phenotypic markers or used for pulmonary organoid construction.

For pulmonary organoid construction, 12 mm cell culture inserts (Merck Millipore, Watford, UK) were first coated with a 60 µL layer of Matrigel and set for 2 h at 37 °C. AFE and mesoderm cells were dissociated in TrypLE (Thermo Fisher Scientific, Loughborough, UK), for 5 min at 37 °C and cells counted. Cell suspensions containing 500,000 cells each of AFE and mesoderm cells per organoid were mixed with an equal volume of Matrigel and 200 µL pipetted onto the first layer of matrigel and set for 2 h at 37 °C. Following this, a final 60 µL layer of Matrigel was pipetted onto the hydrogel and set for 2 h at 37 °C. Finally, cell culture medium, 250 µL DMEM containing Glutamax, non-essential amino acids, human serum replacement 3, BMP4 (10 ng/mL), FGF2 (10 ng/mL), FGF7 (10 ng/mL) and FGF10 (10 ng/mL) were pipetted into the cell culture wells and inserts and incubated at 37 °C. All work was performed with cooled liquids and tips to prevent premature Matrigel polymerisation. Organoids were maintained in culture for up to 14 days and the medium changed every 2–3 days.

### Appendix A.2. qRT-PCR

Total RNA from iPSCs, AFE cells or mesoderm progenitors was prepared using a RNeasy Mini Kit (Qiagen, Hilden, Germany). For total RNA preparation from pulmonary organoids, the organoids-containing Matrigel was placed in 3 mL ice-cold Corning cell recovery solution (Corning, Deeside, UK), which was pipetted up and down to break the Matrigel, and it was incubated on ice for 1 h. The organoids were then collected by centrifugation at 300× *g* for 5 min and incubated with 3 mL 0.05% trypsin/EDTA at 37 °C for 5 min to dissociate cells in the organoids. The cells were collected by centrifugation at 300× *g* for 5 min and then subjected to 1 mL TRIZOL (Sigma Aldrich, Gillingham, UK) and vortexed thoroughly, followed by adding 0.2 mL phenol-chloroform (Sigma Aldrich, Gillingham, UK) and vortexing for 15 s. After incubation for 3 min at room temperature, the sample was centrifuged at 12,000× *g* for 15 min at 4 °C. The upper aqueous phase was carefully collected and transferred into a fresh tube. One half ml isopropanol (Sigma Aldrich, Gillingham, UK) was then added to the sample, which was incubated at room temperature for 10 min followed by centrifugation at 12,000× *g* at 4 °C for 10 min. The RNA pellet was washed twice with 1 mL 75% ethanol and collected by centrifugation at 7500× *g* for 5 min at 4 °C. The RNA pellet was then air-dried and resuspended in 20 μL RNase free H_2_O.

The cDNA was synthesised using an iScript cDNA Synthesis Kit (Bio-Rad Laboratories, Watford, UK) following the manufacturer’s instructions. QRT-PCR reactions containing 5 ng cDNA per sample were prepared using SYBR Green chemistry and pre-designed primer sets (Bio-Rad Laboratories, Watford, UK), as listed in Table A1, and run on a CFX96 Real-Time PCR system (Bio-Rad Laboratories, Watford, UK). Expression was normalised to housekeeping genes.

### Appendix A.3. RNA Sequencing (RNAseq)

Total RNA isolated using the abovementioned method was submitted to the Genomic Technologies Core Facility (GTCF) at the University of Manchester. The quality and integrity of the RNA samples were assessed using a 4200 TapeStation (Agilent Technologies, Cheadle, UK) and then libraries generated using the Illumina^®^ Stranded mRNA Prep. Ligation kit (Illumina, Inc., Cambridge, UK) according to the manufacturer’s protocol. Briefly, total RNA (typically 0.025–1 μg) was used as input material, from which polyadenylated mRNA was purified using poly-T, oligo-attached, magnetic beads. Next, the mRNA was fragmented under elevated temperature and then reverse transcribed into first-strand cDNA using random hexamer primers and in the presence of Actinomycin D (thus improving strand specificity whilst mitigating spurious DNA-dependent synthesis). Following removal of the template RNA, second-strand cDNA was then synthesised to yield blunt-ended, double-stranded cDNA fragments. Strand specificity was maintained by the incorporation of deoxyuridine triphosphate (dUTP) in place of dTTP to quench the second strand during subsequent amplification. Following a single adenine (A) base addition, adapters with a corresponding, complementary thymine (T) overhang were ligated to the cDNA fragments. Pre-index anchors were then ligated to the ends of the double-stranded cDNA fragments to prepare them for dual indexing. A subsequent PCR amplification step was then used to add the index adapter sequences to create the final cDNA library. The adapter indices enabled the multiplexing of the libraries, which were pooled prior to cluster generation using a cBot instrument. The loaded flow-cell was then paired-end sequenced (76 + 76 cycles, plus indices) on an Illumina HiSeq4000 instrument. Finally, the output data was demultiplexed and BCL-to-Fastq conversion performed using Illumina’s bcl2fastq software, version 2.20.0.422 (Illumina, Inc., San Diego, CA, USA).

### Appendix A.4. Flow Cytometry

Cells were dissociated with Accutase for 5 min at 37 °C and then resuspended in HBSS containing Fixable Viability Dye–eFluor 780 (Thermo Fisher Scientific, Loughborough, UK) for 30 min at 4 °C. A sample of cells were retained as an unstained control. Samples were then centrifuged at 300× *g* for 5 min and resuspended in 4% paraformaldehyde (PFA, Sigma-Aldrich, Gillingham, UK) for 4 min. Cells were then diluted 1:1 with PBS containing 2% foetal bovine serum (FBS) (Thermo Fisher Scientific, Loughborough, UK) and centrifuged at 300× *g* for 5 min. Staining for cell surface proteins was performed by resuspending cells in 100 µL PBS with 2% FBS and antibodies found in Table A1 for 30 min at 4 °C. Samples were centrifuged and resuspended as before and permeablised in 100 µL PBS with 2% FBS 0.1% Triton X-100 for 20 min at 4 °C. Following this, staining for intracellular proteins was performed using antibodies listed in Table A1 for 30 min at 4 °C. Finally, samples were centrifuged and resuspended as before, filtered through a 40 µM cell strainer and flow cytometry performed on an LSR II Flow Cytometer (BD Biosciences, Wokingham, UK). Data were analysed with FACSDiva software v8.0 (BD Biosciences, Wokingham, UK), cells were gated to exclude dead cells, debris and cell aggregates, and histograms were drawn to compare the intensity of each fluorophore in stained samples against an unstained control.

### Appendix A.5. Binding of SARS-CoV-2 Spike Protein S1 to Organoids

For the SARS-CoV-2 Spike S1 protein binding to organoids, 5 μL (1 μg) of recombinant SARS-CoV-2 Spike S1 protein (R&D Systems, Abingdon, UK) was added to each well of the organoids culture and incubated in the CO_2_ incubator at 37 °C for 3 h. The organoids were then harvested and imaged by confocal microscopy as described below.

### Appendix A.6. 3D Confocal Imaging

Pulmonary organoids were harvested for immunofluorescent staining and confocal imaging at day 9 after the co-culture of AFE and mesoderm cell populations. Sample preparation was performed as described by Dekkers et al. [32]. The culture medium was first removed, and the organoids were washed with 1 mL of PBS without disrupting the 3D matrix. The entire 3D matrix containing the organoids in the 12 mm cell culture insert was then gently scraped and transferred into a new 24-well plate and plated on ice. One mL of ice-cold cell recovery solution was added to each well and they were then incubated on a horizontal shaker at 4 °C (60 r.p.m.) for 60 min to dissolve the 3D matrix. One-mL tips were pre-coated by dipping the full length of the tips in 1% BSA in PBS. Before using, the tips were used to pipet 1 mL of the BSA solution two times to prevent the organoids from sticking to the tips. The tip was then used to gently resuspend the dissolved organoids-containing matrix five to ten times before transferring the organoids to a 15 mL tube pre-coated with the 1% BSA. The culture well was rinsed with 1 mL of ice-cold 1% BSA to collect all organoids. The 15 mL tube that contains the organoids was topped up to 10 mL with cold (4 °C) PBS and centrifuged at 70× *g* for 3 min at 4 °C to collect the organoids.

For organoid fixation and blocking, the organoids pellet was gently resuspended in 1 mL of 4% paraformaldehyde using a 1 mL tip precoated with 1% BSA, then incubated at 4 °C for 45 min, gently resuspending the organoids halfway through the incubation period. The tube was then topped up to 10 mL with cold (4 °C) 0.1% (vol/vol) PBS-Tween (PBT), gently swirling to mix the sample. After incubation at 4 °C for 10 min, the sample was centrifuged at 70× *g* for 5 min at 4 °C to pellet the organoids. To block the nonspecific binding sites of the organoids, the pellet was first resuspended in cold (4 °C) organoid washing buffer (OWB) that contained 0.1% (vol/vol) Triton X-100 and 0.2% BSA in PBS, and then an appropriate amount (200 μL or more) of organoids suspension per staining was transferred to a low-adherence 24-well plate and left at 4 °C for 15 min.

For immunolabeling, 200 μL of OWB was first added to one of the empty wells, which served as the reference well. The plates containing organoids were tilted to 45° and the OWB were carefully aspirated until 200 μL of OWB with the organoids was left in the plate (the reference well was used to estimate 200 μL). Two hundred μL of primary antibodies in OWB (2× concentration) was added to each well, which were then incubated overnight at 4 °C with mild rocking (60 r.p.m on a horizontal shaker). The following day, 1 mL of OWB was added to each well to wash the organoids. After 3 min, when all organoids were settled at the bottom, the OWB was removed leaving 200 μL in each well. One mL of OWB per well was then added to each well, which were then incubated for 2 h with mild rocking. The washing steps were repeated two more times, then 200 μL secondary antibodies in OWB (2× concentration) were added per well and incubated at 4 °C overnight with mild rocking. The washing steps were repeated at least three times. The organoids were then counterstained by incubating with 0.1 μg/mL DAPI (Thermo Fisher Scientific, Loughborough, UK) for 15 min under dark at RT, and then the above washing steps were repeated three times, with a final rinsing with PBS (containing 0.1% BSA). The organoids were then ready for imaging.

For 3D imaging, the organoids were transferred to a 35 mm µ-Dish (ibidi, Gräfelfing, Germany) pre-filled with 3 mL 1% BSA in PBS. The organoid suspension was gently swirled to collect the organoids to the centre of the dish and allowed to settle for 5 min before being imaged on a Leica SP8 confocal microscope with 20× water immersion objective for multiphoton imaging. The acquisition settings were: scan mode = sequential scan, format = 1024 × 1024, speed = 600 HZ, line average = 3.0, line accumulation 1.0, frame average 1.0, frame accumulation1.0, rotation 0.0, pinhole 1AU, zoom factor 2.0~3.0, zoom factor 2.0~3.0. Seq.1 was assigned to detect DAPI and AF647, using wavelengths of 410–478 nm and 657–800 nm, respectively. Seq.2 was assigned to detect AF488 (498–637 nm) and the white light. The Hybrid detector (HyD) was chosen to detect fluorescent signals. Smart gain for PMT was set to 800–960, smart gain for HyD was set to 10–40%, and the smart offset was 0.0%. The laser% for all fluorescence was ranging from 5% to 15% to gain an optimum imaging.

### Appendix A.7. Histology

Human lung sections were obtained following informed consent and ethical approval (National Research Ethics Service, REC 20/NW/0302) from the Manchester Allergy, Respiratory and Thoracic Surgery (ManARTS) Biobank (study number M2014-18). Immunofluorescent staining of 5 µm sections of human lung was performed as follows. Samples were deparaffinised and rehydrated through sequential washes with xylene and 100% and 90% ethanol, 3 min each. Antigen retrieval was performed in boiling citrate buffer, pH6, for 20 min, after which samples were incubated with primary antibodies (listed in the Key resource table) overnight at 4 °C. The next day, samples were washed 3 times with PBS and then incubated with secondary antibodies, Table A1, for 2 h at room temperature. Finally, samples were washed 3 times with PBS, dehydrated through 70%, 90% and 100% ethanol, and mounted with ProLong Diamond Antifade Mountant containing DAPI (Thermo Fisher Scientific, Loughborough, UK). Slides were imaged on an Olympus IX83 inverted microscope and images processed with Olympus CellSens software v1.16 (Evident, Tokyo, Japan).

### Appendix A.8. Statistics and Data Analysis

Statistical analysis: Statistical significance was determined by unpaired Student’s *t*-test using GraphPad Prism. Data are presented as mean ± SEM. A minimum significance value of *p* < 0.05 was considered significant.

RNAseq analysis: Unmapped paired-end sequences from the HiSeq 4000 were assessed by FastQC (http://www.bioinformatics.babraham.ac.uk/projects/fastqc/, accessed on 15 October 2021). Sequence adapters were removed, and reads were quality trimmed to quality q20 using Trimmomatic_0.36 (PMID: 24695404). The reads were mapped against the reference human (hg38) genome and counts per gene were calculated using annotation from GENCODE 30 (http://www.gencodegenes.org/, accessed on 15 October 2021) using STAR_2.5.3a (PMID: 23104886). Normalisation, principal components analysis (PCA) and differential expression were calculated in DESeq2_1.20.0 using default settings (PMID:25516281) and independent filtering significance level (alpha) 0.05. Gene Ontology enrichment analysis was conducted using the clusterProfiler software package (version 3.16.0; Bioconductor, Boston, MA, USA) to visualise biological functions enriched in the differentially expressed and notable upregulated (>2-fold mean increase) or downregulated (>2-fold mean decrease) genes of the dataset. The lung-differentiation-related genes were re-identified and presented through heatmap plot with pheatmap software package (version 1.0.12; R Foundation for Statistical Computing, Vienna, Austria).

**Table A1:** Reagent details.

Reagent	Supplier	Catalogue Number
ACE2 primary antibody	R&D Systems	MAB933-SP
AQP5 primary antibody	ThermoFisher Scientific	PA5-99403
SFTPA1 primary antibody	Universal Biologics	A3133-50ul
SFTPB primary antibody	Biomatik	CAU25609
SFTPC primary antibody	ThermoFisher Scientific	PA571680
T1α primary antibody	ThermoFisher Scientific	BMS1105
TTF1 primary antibody	Caltag Medsystems	H00007080-M01-100ug
Donkey anti Mouse A488 secondary antibody	Abcam	ab150105
Donkey anti Rabbit A594 secondary antibody	Abcam	ab150076
FOXA2 e660 conjugated antibody	eBioscience	50-4778
GATA4 A647 conjugated antibody	Bioss	bs-1778R-A647
PDGFRa A594 conjugated antibody	Bioss	bs-0231R-A594
SOX17 PerCP CY5.5 conjugated antibody	BD Pharmingen	562387
TBX1 A488 conjugated antibody	Bioss	bs-8257R-A488
VEGFR2 PE conjugated antibody	Bioss	bs-10412R-PE
*FOXA2* primer	BioRad	qHsaCID0014658
*GATA4* primer	BioRad	qHsaCID0012121
*PDGFRa* primer	BioRad	qHsaCID0007202
*SOX17* primer	BioRad	qHsaCED0046246
*TBX1* primer	BioRad	qHsaCID0013392
*VEGFR2* primer	BioRad	qHsaCID0006310
*B2M*	Forward: 5′ GAGGCTATCCAGCGTACTCCA 3′	Reverse: 5′ CGGCAGGCATACTCATC TTTT 3′
*GAPDH*	Forward: 5′ CATGTTCGTCATGGGTG TGAACCA 3′	Reverse: 5′ ATGGCATGGACTGTGGT CATGAGT 3′
Recombinant SARS-CoV-2 Spike S1 protein	R&D Systems	10522-CV
SARS-CoV-2 Spike S1 Subunit Antibody	R&D Systems	MAB105403-SP

## Appendix C. Gene Ontology Terms of Lung-Development-Related Gene Sets

Lung-specific genes: *IGFBP5*, *WNT5A*, *WNT7B*, *EGFR*, *LTBP3*, *EPAS1*, *SOX9*, *TGFB3*, *ITGB6*, *NOG*, *TNC*, *NODAL*, *THRB*, *RIDA*, *ADAMTS9*, *LOX*, *GPC4*, *TIMELESS*, *STRA6*, *PDGFRA*, *ABCA12*, *KLF2*, *ASXL1*, *CRISPLD2*, *WNT11*, *ALDH1A2*, *EIF4E*, *WNT5A*, *FOXF1*, *SRF*, *GATA6*, *GPC5*, *ZIC3*, *FGF2*, *CELSR1*, *NPHP3*, *PDPN*, *ADAMTS16*, *GPC3*, *CCN2*, *FGF7*, *MMP14*, *CSF2RB*, *RTKN2*, *CEBPA*, *HMGB1*, *PLVAP*, *SAV1*, *HSPG2*, *LRRN4*, *TBX4*, *FOXA1*, *LAMP3*, *PROS1*, *PROX1*, *FGF10*, *SPARC*, *FGF8*, *TCF21*, *PTN*, *ADA*, *ADAMTS2*, *FSTL3*, *ACOXL*, *NFIB*, *WNT2*, *PKD1*, *VEGFA*, *RDH10*, *CYP2F1*, *ATP7A*, *HES1*, *RCN3*, *NOTCH1*, *WNT2B*, *TNF*, *TTF1*, *SLC6A16*, *SLC6A15*, *STK40*, *CDKN1A*, *HOXA5*, *BMP4*, *BMPR2*, *RSPO2*, *SIM2*, *ZFPM2*, *MAP2K1*, *PDGFRB*, *CP*, *TBX5*, *GLI3*, *ACVR2B*, *FLT4*, *FOXJ1*, *GRHL2*, *AIMP2*, *AGER*, *ETV5*, *THRA*, *TGFBR2*, *AARD*, *GPC2*, *LAMA5*, *SFTPA2*, *AQP5*, *SFTPD*.

Lung development: *PDPN*, *STK40*, *WNT2B*, *PROX1*, *EPAS1*, *ITGB6*, *BMPR2*, *ABCA12*, *IGFBP5*, *THRB*, *TGFBR2*, *WNT5A*, *NPHP3*, *HES1*, *PDGFRA*, *EIF4E*, *FGF2*, *FGF10*, *LOX*, *PDGFRB*, *SPARC*, *ADAMTS2*, *FLT4*, *TNF*, *SRF*, *VEGFA*, *CCN2*, *TCF21*, *AIMP2*, *HOXA5*, *GLI3*, *EGFR*, *WNT2*, *PTN*, *RDH10*, *GRHL2*, *ZFPM2*, *RSPO2*, *NFIB*, *TNC*, *NOTCH1*, *NODAL*, *FGF8*, *LTBP3*, *WNT11*, *TIMELESS*, *TBX5*, *HMGB1*, *MMP14*, *FOXA1*, *SAV1*, *BMP4*, *TGFB3*, *FGF7*, *ALDH1A2*, *MAP2K1*, *STRA6*, *PKD1*, *CRISPLD2*, *FOXF1*, *THRA*, *NOG*, *TBX4*, *SOX9*, *FOXJ1*, *GATA6*, *FSTL3*, *KLF2*, *CEBPA*, *RCN3*, *ASXL1*, *ADA*, *LAMA5*, *SIM2*, *WNT7B*, *CELSR1*, *ATP7A*, *GPC3*, *ZIC3*.

Lung epithelium development: *THRB*, *FGF10*, *AIMP2*, *HOXA5*, *WNT2*, *GRHL2*, *NFIB*, *FOXA1*, *SAV1*, *BMP4*, *FGF7*, *MAP2K1*, *STRA6*, *PKD1*, *THRA*, *SOX9*, *FOXJ1*, *GATA6*, *KLF2*, *RCN3*, *WNT7B.*

Lung morphology: *PIK3CD*, *WNT4*, *GRHL3*, *CLIC4*, *STIL*, *TACSTD2*, *GLMN*, *CSF1*, *WNT2B*, *PROX1*, *TGFB2*, *KIF26B*, *OSR1*, *RHOB*, *SIX2*, *TBR1*, *LRP2*, *BBS5*, *HOXD11*, *WNT6*, *IHH*, *EPHA4*, *GBX2*, *TGFBR2*, *TRIM71*, *WNT5A*, *HESX1*, *FOXP1*, *CASR*, *NPHP3*, *DVL3*, *OPA1*, *HES1*, *KDR*, *SHROOM3*, *NPNT*, *LEF1*, *FGF2*, *FAT4*, *SFRP2*, *HAND2*, *CASP3*, *IRX1*, *ADAMTS16*, *GDNF*, *FGF10*, *FOXD1*, *MEF2C*, *CSF1R*, *HAND1*, *NKX2-5*, *SRF*, *VEGFA*, *BMP5*, *TCF21*, *SOSTDC1*, *TWIST1*, *HOXA5*, *HOXA11*, *GLI3*, *COBL*, *WNT2*, *PODXL*, *GATA4*, *DLC1*, *NKX3-1*, *SFRP1*, *SOX17*, *RDH10*, *GRHL2*, *FZD6*, *CTHRC1*, *RSPO2*, *MTSS1*, *MYC*, *FOXH1*, *TNC*, *ENG*, *NOTCH1*, *GATA3*, *NRP1*, *RET*, *NODAL*, *KIF20B*, *PAX2*, *FGF8*, *SUFU*, *WT1*, *MDK*, *WNT11*, *PAK1*, *ACVRL1*, *TIMELESS*, *LGR5*, *CEP290*, *TCTN1*, *TBX3*, *KDM2B*, *STARD13*, *MMP14*, *NFATC4*, *FOXA1*, *BMP4*, *SIX1*, *MTHFD1*, *GREM1*, *DLL4*, *SMAD3*, *BBS4*, *MESP1*, *PKD1*, *SALL1*, *IRX3*, *FOXF1*, *FOXC2*, *ALOX12*, *CNTN1*, *MED1*, *WNT9B*, *HOXB7*, *NOG*, *TBX2*, *SOX9*, *PRKACA*, *TGFB1*, *RASIP1*, *FUZ*, *BMP2*, *OVOL2*, *PSRC1*, *SALL4*, *LAMA5*, *CECR2*, *CELSR1*, *LRP5L*, *PLXNB2*, *AGTR2*, *GPC3*, *ZIC3.*
